# Transinusal Pathway Removal of an Impacted Third Molar with an Unusual Approach: A Case Report and a Systematic Review of the Literature

**DOI:** 10.3390/antibiotics11050658

**Published:** 2022-05-13

**Authors:** Luan Mavriqi, Felice Lorusso, Gianluca Tartaglia, Francesco Inchingolo, Antonio Scarano

**Affiliations:** 1 Faculty of Dental Medicine, Albanian University, 1023 Tirane, Albania; luanmavriqi@yahoo.com; 2 Department of Innovative Technologies in Medicine & Dentistry, University of Chieti-Pescara, 66100 Chieti, Italy; drlorussofelice@gmail.com; 3 Department of Biomedical, Surgical and Dental Sciences, School of Dentistry, University of Milan, 20122 Milano, Italy; gianluca.tartaglia@unimi.it; 4 Department of Interdisciplinary Medicine, University of Bari “Aldo Moro”, 70121 Bari, Italy; francesco.inchingolo@uniba.it; 5 Department of Oral Implantology, Dental Research Division, College Ingà, UNINGÁ, Cachoeiro de Itapemirim 29312, Espirito Santo, Brazil

**Keywords:** osseointegrated implant, nasal cavity, endoscopy procedure, fixture displacement, ambulatory surgical setting, day surgery, device suction, endoscopy procedure, forceps

## Abstract

Background: The purpose of the present case report was to investigate a very rare ectopic third molar removal by a trans-sinusal approach and report the study findings through a systematic review of the literature on this topic. Case presentation: A 38-year-old female patient was visited for pain at the level of the right maxillary region. No relevant medical history was reported. The CBCT tomography assessment revealed an impacted third tooth at the level of the postero-lateral maxilla. Review Methods: An electronic search was performed through Boolean indicators query on Pubmed/Medline, EMBASE, Cochrane Library databases. The clinical reports were identified and selected in order to perform a descriptive analysis. The surgical approach concerned a trans-sinusal access to the site for the ectopic tooth removal with a lateral antrostomy. No evident inflammatory alteration was associated to the ectopic tooth and a non-relevant post-operative sequelae was reported at the follow up. Results: A total of 34 scientific papers were retrieved from the database search. Only two cases reported a wait-and-see radiographical follow-up approach, while the most common treatment was surgical removal, also for asymptomatic cases. Conclusion: The third-molar ectopic tooth into the maxillary sinus is an uncommon occurrence that is beast treated by an in-chair intraoral tran-sinusal approach, with a consistent reduction of the invasivity, a mild morbidity and a successful functional outcome.

## 1. Introduction

Problems with mandibular and maxillary third molars together with maxillary canines are frequently observed in oral surgery and their extraction is one of the most widespread procedures in oral surgery. Clinical and radiologic evaluation of surgical difficulty can help the practitioner plan the surgical technique for avoing the possible complications. Different surgical techniques has been proposed for maxillary ectopic teeth that described trans-oral and Caldwell-Luc approaches, extra-oral accesses [1], nasal endoscopic assisted techniques [2], that take advantage to the teeth clinical presentation and localization in the paranasal cavities. The latter approach has the advantage of visualization of potential orbital floor defects. In the present paper, we report a case of headache, facial pain associated with a retained molar within the maxillary sinus.

Usually, the diagnosis of impacted third molars is performed during the first dentistry visit or during primary care. The extraction of a maxillary wisdom tooth involves the risk of complications. The most frequent complications reported are the displacement in the maxillary sinus [3], oroantral communication [4], displacement into the lateral buccal space [5], into infratemporal fossa [6]. The displaced tooth in the maxillary sinus or oral antral communication are the most frequent complications due to the intimate relationship between the wisdom tooth and the maxillary sinus tooth [7]. The purpose of this case report is to describe the trans-sinusal approach used to remove an impacted maxillary third molar and review the scientific literature regarding the recurrency of the ectopic molars in sinus cavity, the drugs administration and the techniques adopted.

## 2. Case Report

The present study was conducted in accordance to the ethical laws and the World Medical Association Declaration of Helsinki [8] and the Surgical Case Report (SCARE) guidelines [9].

**Figure 1:**
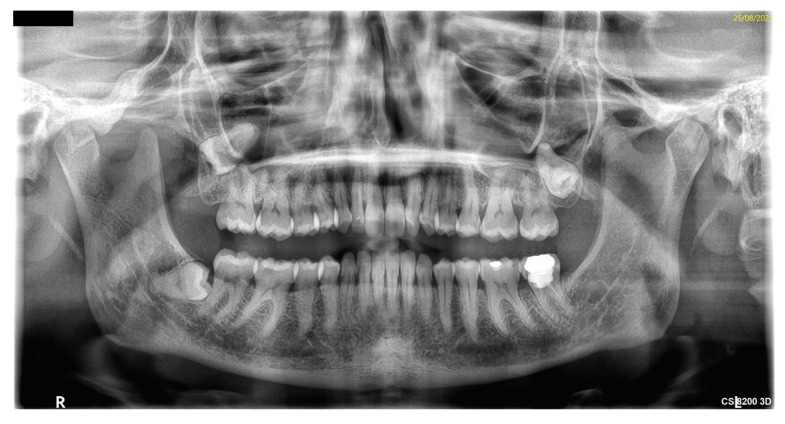
Impacted symptomatic 18 on OPG.

A 38-year-old female had chronic pain in the maxillary right region and was diagnosed with having an impacted third tooth by orthopantomogram (OPG) (Figure 1).

**Figure 2:**
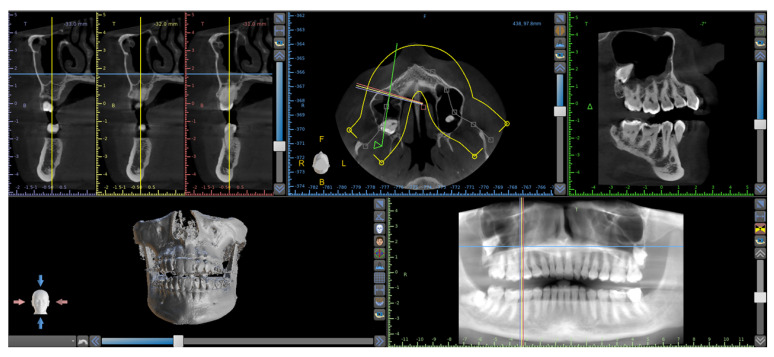
Coronal section shows wisdom tooth much higher with part of the crown and all part of the roots in the distal wall of the sinus maxillary and above the second molar.

Clinical examination revealed absence of the right maxillary third molar, radiologic examination excluded the presence of carious or periodontal disease borne by neighboring teeth (Figure 2). The subject did not report a history of restorative or prosthetic treatment procedures performed in the recent years. After clinical examination a Cone Beam Computed Tomography (CBCT) (Vatech Ipax 3D PCH-6500, Fort Lee, NJ, USA) was executed to assess presence of carious, periodontal disease or impacted tooth. The radiograph showed an impacted wisdom without an enlarged follicle much higher with part of the crown and all part of the roots in the distal wall of the sinus maxillary and above the second molar (Figure 2).

**Figure 3:**
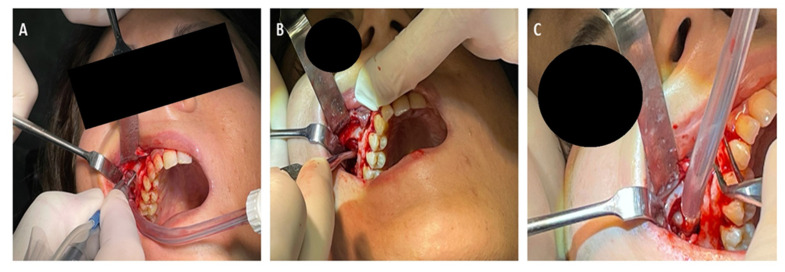
A modified triangular flap without anterior release was performed for access to the sinus maxillary (**A**). A window by rosette round bur was opened in the posterior part of the maxillary sinus (**B**). After dislocation the wisdom tooth was aspirated (**C**).

After explaining the different surgical possibilities to the patient, we decided to perform a transinusal access. The wisdom extraction was scheduled in an ambulatory setting and under local anesthesia. Prior to surgical treatment, the patient’s mouth was rinsed with a chlorhexidine 0.2% digluconate solution (Curaden Healthcare S.p.A., Saronno, Italy) for 2 min. The local anesthesia was performend by the administration of Articaine^®^ (Ubistesin 4%-Espe Dental AG, Seefeld, Germany) with epinephrine of 1:100,000.. Written informed consent was acquired from the patient for publication of the case report. A modified triangular flap without anterior release was performed as previously described for access to the sinus maxillary [10,11,12]. A window was opened by rosette round bur in the posterior part of the maxillary sinus (Figure 3).

The tooth was observed in the posterolateral aspect of the maxillary sinus, no bone was present around the dental crown and no pericoronal lesion was detected. The maxillary sinus was entered through the Schneiderian membrane and the tooth was dislocated with a lever and then grasped with a klemmer and removed.

The maxillary sinus window was covered by pericardium membrane (Shelter slow, Ubgen, Padova, Italy) [11]. The flap was carefully sutured with Polimid 4.0 (Assut, Magliano de’ Marsi, Italy), which was removed after seven days. An analgesic medication (ibuprofen 600 mg) 2 h following surgery and every 6 h afterward was prescribed, to be continued for 3 days. Also, a single dose of betamethasone 4 mg was given to limit postoperative edema of the face and cheek, and the patient was told not to blow her nose for two weeks to avoid mouth-antral communication. No complication was reported during post operative time. After 1 month the patient describes the disappearance of chronic pain in the right maxillary region.

## 3. Systematic Review Methodology

### 3.1. Search Strategies

Screening of the articles was performed in accordance to the Standards for Reporting Qualitative Research principles (SRQR) and the “Preferred Reporting Items for Systematic Reviews and Meta-Analyses” (PRISMA) guidelines. The search strategy was conducted on a keywords search strategy as described in Table 1. The initial screening was performed by PubMed/MEDLINE, EMBASE and Cochrane electronic databases (28 January 2022) following the Boolean indicators described in Table 1. The title and abstracts of the scientific studies was evaluated and an initial screening was performed. The articles were limited to randomized and non-randomized human clinical trials, prospective and retrospective studies, clinical case reports and case series with ectopic maxillary third molar into the sinus antrum and the full text was evaluated to conduct the eligibility to the descriptive analysis Table 2.

### 3.2. Inclusion and Exclusion Criteria

The criteria necessary for inclusion in the qualitative synthesis were limited to human clinical trials, prospective and retrospective studies with a minimum follow up of 1 month with no restriction of surgical technique, alternative medical approach, post-operative sequelae, and number of protocol stages. The exclusion criteria were systematic and literature reviews, letters to the editor, and in vitro and laboratory simulation. The articles written in non-English language were eliminated from the assessment.

### 3.3. Article Identification Procedure

The eligibility assessment was conducted independently by two expert reviewers (AS, FL). Also, a manual search was conducted to increase the article pool for the full-text assessment. The articles written in English that satisfied the inclusion criteria were considered while the duplicates and the articles excluded were categorized reporting the exclusion reasons.

### 3.4. Study Assessment

The data of the articles was evaluated independently by the reviewers using a special designed electronic form according to the following categories: study model design, patient’s age, major symptoms, neoplasms or associated neoformations, treatment protocol, surgical technique, post-operative sequelae, position of the teeth, number of subjects and study findings (Table 2). The peri-operative drugs administration has been recorded and discussed.

## 4. Literature Review Results

### 4.1. Paper Selection: General Characteristics

**Figure 4:**
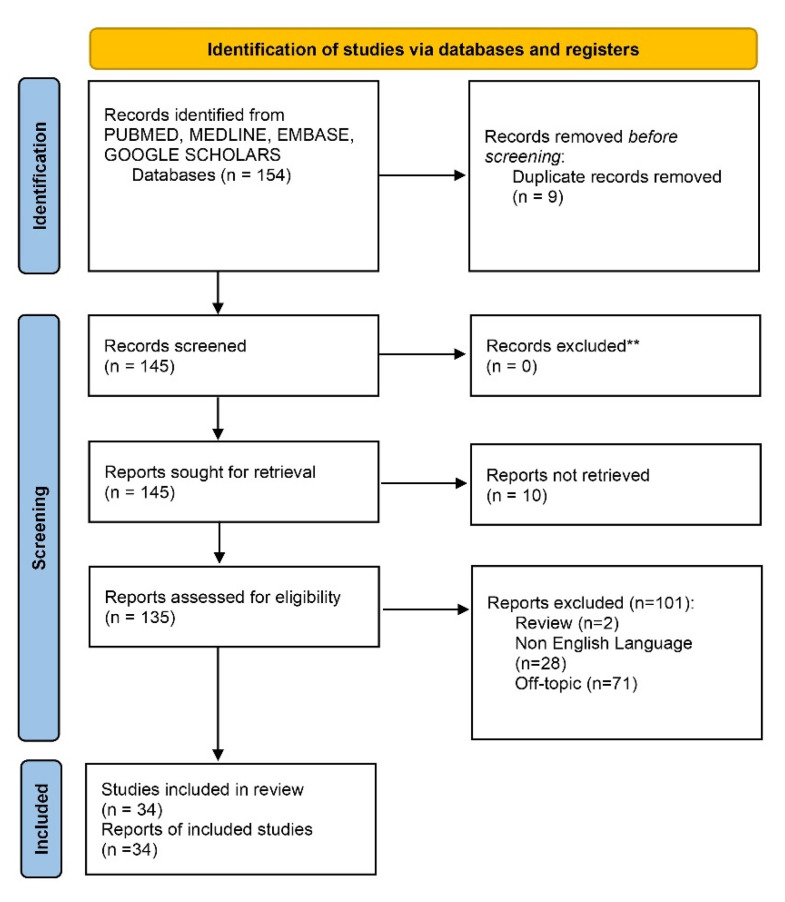
Prisma flowchart of the database search and study retrieval process.

The manuscript screening and eligibility, as presented in Figure 1, followed the PRISMA guidelines. The electronic database and manual search output retrieved a total of 154 manuscripts, although a total of 9 duplicates were removed. After the initial screening, a total of 145 manuscript were considered but the full text of 10 papers was not available. The eligibility assessment was conducted on 135 articles and 2 reviews, 71 off-topic papers, 28 non-English papers. A total of 34 articles were selected for the qualitative synthesis (Figure 4).

### 4.2. Included Study Characteristics

The review selection included a total of 6 case series and 28 case reports for a total of 78 patients, 81 cases and 3 bilateral. The patients’ ages were between 8 years old and 61 years old. No clinical trial/randomized clinical trial related to the review search topic were identified. A total of 12 papers reported asymptomatic cases [13,14,15,16,17,18,19], while the most common main symptoms were chronic nasal obstruction [20,21], bilateral rhinorrhea [20,36,37,47], pus discharge [21,30,31,32,33,34,42], sinusitis [22,29,40,42,44,45], pain and swelling [23,33,35,36,37]. A total of 26 cases reported an associated dentigerous cyst [13,16,17,21,22,24,29,30,32,33,35,38,39,40,42,43,44,45], 2 cases of associated inflammatory cyst [25,26] and 1 case of associated osteoma [41]. In 2 subjects a “wait and see” approach was followed with a radiographical follow up very year [17,31]. In 11 cases an endoscopic approach was performed [13,14,19,21,26,27,28,29,40,41,42], while a total of 21 cases received a Caldwell-Luc antral approach [14,16,22,24,25,26,27,28,30,32,33,34,35,36,37,38,39,41,43,44,46]. The post-operative sequelae were often associated to a mild healing symptoms, while in some cases peri-orbital emphysema, transient cheek numbness and bleeding was present [13]. Referring to the wisdom molar position, the most frequent presentation was associated to the inferior and inferomedial wall [13,14,29,44]. Less frequent were the orbital wall presentation [13,16,19,38], the sinus floor [13,44], medial and postmedial wall [20,22,26,28,29,32,33,35,36,39,40,42].

### 4.3. Drug Administration Protocols

Very few information has been detected concerning the peri-operative antibiotics and analgesic protocols in the included studies. An heterogeneous antibiotics prophylaxis administration was reported while the most common were amoxicillin [23,46], amoxicillin combined with clavulanate potassium [26]. The most frequently administered analgesic therapy or non-steroidal anti-inflammatory substance were [16,19] diclofenac sodium [23], acetaminophen [46], nimesulide, and paracetamol [26]. Prolonged nasal decongestion therapy was adopted to reduce post-operative sinusitis occurrence [20].

## 5. Discussion

In the present case report we describe the clinical characteristics of a patient with a wisdom tooth in the maxillary above the second molar that which caused chronic pain. Maxillary third molar extraction is a frequent surgical intervention in oral and maxillofacial surgery. As also evinced by the review of the literature, in this particular case the clinical presentation of the ectopic tooth was very rare and associated to the postero-lateral wall of the maxilla. In fact, the rationale of the present investigation was to contextualize the literature recurrency of this particular clinical condition and support a transinusal approach for impacted upper third molar. Many factors can influence ectopic tooth, including trauma presence of benign or malignant lesions including rhinogenic and odontogenic infections or iatrogenic activity [48,49]. The present case report represents a very rare case of a wisdom tooth producing pain without local sinus symptoms and inflammation. We decided to extract the tooth by transinusal pathway removal because this approach was found to be simpler and avoided all lesion of the second molar and for the high risk of displacement of the tooth into the maxillary sinus. In this case we would still have had to access the maxillary sinus to remove the tooth. Moreover, the transinusal approach is more invasive but the visibility is considerably better. However, this type of approach could be complicated by sinusitis or an oroantral fistula. In literature, a total of 34 cases were retrieved from the systematic analysis that reported having ectopic wisdom teeth in the maxillary sinus that can cause symptoms such as headache, facial pain, sinusitis or swelling, nasal obstruction, rhinorrhea compressing the nasolacrimal canal [50,51] or can be completely asymptomatic [48,52]. According to the present systematic search, the age of diagnosis is very heterogeneous and symptoms-correlated, while the most common presentation of ectopic upper third molar was associated to a posterior wall (24.13%), the lateral wall (22.41%), the medial wall (18.96%), inferior wall (17.24%), orbit floor (15.51%). The ectopic third molar is relatively rare at the level of the antrum anterior wall (1.72%). According to the drug prophylaxis, the antibiotics therapy is often administered through amoxicillin or amoxicillin/clavulanate combination associated to non-steroidal anti-inflammatory protocol [23,46]. The adopting of a prolonged nasal decongestion therapy is useful to reduce post-operative sinusitis sequelae. The using of corticosteroid is not always prescribed in literature [20]. The patient presented with headache, facial pain without inflammatory pericoronal lesion sign or sinusitis, that were confirmed by CBCT. In the case presented, a single dose of betamethasone was administered to avoid local edema, while no complications were reported during healing period with a complete recovery time of 1 month from the surgery. In the literature different approaches were proposed for managing ectopic teeth in the maxillary, such as extra and trans-oral approaches [1] or endoscopic assisted procedures [2], or the endoscopically assisted Caldwell-Luc approach [53,54]. The latter approach has the advantage of visualization of potential orbital floor defects. In the present paper, we report a case of headache, facial pain associated with a retained molar within the maxillary sinus.

## 6. Conclusions

In conclusions, the ectopic teeth localization could deeply influence the clinical manifestation, symptoms and the surgical approach to reduce the post-operative morbidity and complications sequelae. In order provide a useful guidance for surgeons and dentists for the management of ectopic wisdom in clinical practice, the transinusal pathway approach combined with the antibiotic prophylaxis and corticosteroid administration could reduce the post-operative symptoms reducing the face edema and accelerate the recovery period.

## Figures and Tables

**Table 1:** Electronic database boolean search: keyword strategy.

	Search Strategies
Keywords:	Advanced keywords search: ((ectopic OR wisdom OR Third molar) AND teeth AND maxilla* sinus))
Databases	*Pubmed/Medline, EMBASE, Cochrane Library*

**Table 2:** Articles selection descriptive synthesis.

Authors	Journal	Study Design	Age	Principal Symptoms	Lesions	Treatment Protocol	Technique	Post Operative Sequelae	Drug Therapy	Position	Subject(s)	Study Findings
Masalha et al. [13]	J Clin Exp Dent	Case series	Range 16–61 yo	(1) Sinonasal symptoms (7), (2) asymptomatic (2), (3) oro-antral fistula (1)	dentigerous cyst (8)	Lesion enucleation/exodontia	transnasal endoscopic approach (TEA)	(a) self-limited periorbital emphysema (1) (b) transient cheek numbness (1) (c) early post-operative bleeding (1)	-	(a) Inferior wall (*n* = 1) (b) Inferior/Medial wall (*n* = 2) (c) Orbital floor (*n* = 3) (d) Floor (*n* = 3) (e) Anterior/medial wall (*n* = 1) (f) Lateral wall (*n* = 1)	10 subjects (11 cases)	Trans-nasal Endoscopic surgery is feasible and safe
Allen et al. [14]	J Surg Case Rep	Case report	14 yo	asymptomatic	-	Exodontia	Caldwell–Luc endoscopic approach with maxillary antrostomy	Postoperative period unremarkable.	-	Posterior inferior maxillary sinus	1 subject	The transoral removal of the tooth in pediatric subject with no anterior nasal trauma.
Yagiz et al. [15]	J Stomatol Oral Maxillofac Surg.	Case report	38 yo	asymptomatic	-	Exodontia	Intraoral exodontia	Postoperative period unremarkable.	-	Lateral wall	1 subject	The tooth evidence on the OPT was ghost image of the impacted distomolar at the contralateral side
Balaji et al. [16]	Indian J Dent Res	Case report	42 yo	asymptomatic	dentigerous cyst	Exodontia	Intraoral Caldwell Luc procedure	Postoperative period unremarkable.	Antibiotic coverage and non-steroidal anti-inflammatory drugs were administered for 5 days	Orbit floor	1 subject	An ectopic distomolar along the floor of the orbit with dentigerous cystic features is rare.
Liu et al. [17]	J Int Med Res	Case report	6 yo	asymptomatic	dentigerous cyst	No intervention	-	Postoperative period unremarkable.	-	Posterior wall	1 subject	A congenital bone defect was observed in the posterior wall of the maxillary sinus
Lai et al. [18]	J Radiol Case Rep	Case report	52 yo	asymptomatic	-	Exodontia	Intraoral maxillary antrostomy	Postoperative period unremarkable.	-	posterolateral wall	1 subject	Ectopic tooth in the maxillary sinus is a rare condition
Viterbo et al. [19]	J Craniofac Surg	Case report	29 yo	asymptomatic	-	Exodontia	endoscopic sinus surgery	Postoperative period unremarkable.	Antibiotics was administered from the day before the surgery and prolonged for 5 days postoperatively. Corticosteroid and analgesic administration was provided.	Floor of the orbit	1 subject	If untreated, this occurrenc could determine infections or develop a cyst
Lombroni et al. [20]	Indian J Dent Res	Case report	37 yo	chronic nasal obstruction and moderate bilateral rhinorrhea	-	radiology follow-up	-	-	Prophylaxis associated to prolonged nasal decongestion therapy was administered	Medial wall	1 subject	The follow up approach is mainly for asymptomatic patients, preferring periodic checks every 6–8 months
Liau et al. [21]	J Craniofac Surg	Case report	63 yo	chronic nasal obstruction with associated intraoral discharge. Tuber chronic oroantral fistula	dentigerous cyst	Lesion enucleation/exodontia	endoscopic-assisted middle meatal antrostomies surgery	Postoperative period unremarkable.	-	Posterosuperior wall	1 subject	The combined technique with of endoscopic visualisation for a Caldwell-Luc approach
Datli et al. [22]	J Craniofac Surg	Case report	41 yo	Chronic Sinusitis	dentigerous cyst	Lesion enucleation/exodontia	Caldwell-Luc maxillary antrostomy/Penrose drain	Postoperative period unremarkable.	-	posteromedial-lateral walls	1 subject	Dentigerous cysts caused by an ectopic tooth should be resected
Rai et al. [23]	Indian J Dent Res	Case report	46 yo	complaints of watering from the left eye, pain and swelling	-	Exodontia	Intraoral maxillary antrostomy	Postoperative period unremarkable.	Amoxicillin (500 mg × 3/day) for a week and diclofenac sodium (50 mg × 2/day) were administered for 5 days	Lateral/superior wall	1 subject	Intraoral approach is superior to the extraoral and endoscopic approach
Kara et al. [24]	J Istanb Univ Fac Dent	Case report	16 yo	expansile swelling	dentigerous cyst	Lesion enucleation/exodontia	Marsupialization, Caldwell-Luc maxillary antrostomy (2 stage surgery)	Postoperative period unremarkable.	-	Posterosuperior wall	1 subject	Marsupialization and enucleation was preferred for treatment, because of size of cyst and anatomical relationship of tooth.
Touiheme et al. [25]	Pan Afr Med J	Case report	23 yo	Facial pain, chronic synusitis and mucopurulent rhinorrhoea	Inflammatory cyst	Lesion enucleation/exodontia	Caldwell-Luc maxillary antrostomy	Postoperative period unremarkable.	-	Lateral wall	1 subject	The extraction can be performed by conventional approach or by transnasal endoscopic approach with less morbidity.
Chagas Júnior et al. [26]	Craniomaxillofac Trauma Reconstr	Case report	60 yo	Facial trauma (age 6), claiming breathing and phonatory difficulties	Inflammatory cyst	Exodontia	combined approach of endoscopic sinus surgery and Caldwell-Luc procedure	recurrent oroantral fistula.	Amoxicillin Clavulanate Potassium (875 mg + 125 mg) each 12 h for two weeks, Nimesulide (100 mg) every 12 h for 4 days, paracetamol (750 mg) every 6 h in case of pain or fever	Medial/superior wall	1 subject	The Caldwell-Luc was chosen to remove the cyst adjacent
Saleem et al. [27]	Head Face Med	Case report	45 yo	haemoptysis.	-	Exodontia	endonasal endoscopic uncinectomy and Caldwell-Luc maxillary antrostomy	Postoperative period unremarkable.	-	Lateral- superior wall	1 subject	Presence of foreign bodies and ectopic teeth in paranasal sinuses can be reliably excluded
Topal et al. [28]	Turk Arch Otorhinolaryngol	Case report	32 yo	left buccal mucosa was diffusely edematous.	-	Exodontia	combined approach of endoscopic sinus surgery and Caldwell-Luc procedure	Postoperative period unremarkable.	-	Medial wall	1 subject	Surgical removal is required for complicated patients.
Abd El-Fattah et al. [29]	Clin Otolaryngol	Case series	Range 28–48 yo	Loosening of tooth, Sinusitis, Ocular pain	dentigerous cyst	Lesion enucleation/exodontia	(1) Endoscopic-assisted transoral sublabial approach (2) Endoscopic endonasal through middle meatal antrostomy (3) Endoscopic endonasal pre-lacrimal approach	Postoperative period unremarkable.	-	(a) Inferior wall (*n* = 5) (b) Superior wall (*n* = 2) (c) Medial wall (*n* = 2) (d) Lateral wall (*n* = 2)	11 subjects	Ectopic teeth associated with cysts in the could be removed with the endoscopic/endoscopic-assisted approaches
Guruprasad et al. [30]	J Clin Imaging Sci	Case report	21 yo	nasal discharge of pus, Swelling	dentigerous cyst	Lesion enucleation/exodontia	Caldwell-Luc maxillary antrostomy	Postoperative period unremarkable.	-	Lateral wall	1 subject	Management of dentigerous cyst arising from ectopic third molar in the maxillary sinus is usually enucleation.
Di Felice et al. [31]	Aust Dent J	Case report	35 yo	nasal discharge of pus, Swelling	-	radiology follow-up	-	Solved through antibiotic theraphy	-	Postero-lateral wall	1 subject	Treatment of ectopic teeth in the maxillary sinus is usually surgical extraction.
Kasat et al. [32]	Contemp Clin Dent.	Case report	22 yo	nasal pus discharge	dentigerous cyst	Lesion enucleation/exodontia	Incisional biopsy, Caldwell-Luc procedure	Postoperative period unremarkable.	-	posteromedial-lateral walls, Oro-antral fistula	1 subject	Occurrence of an ectopic tooth in the maxillary sinus and association of a dentigerous cyst with it is a rare phenomenon.
Sharma et al. [33]	Int J Surg Case Rep	Case report	27 yo	nasal discharge and facial pain	dentigerous cyst	Lesion enucleation/exodontia	Intraoral Caldwell Luc procedure	Postoperative period unremarkable.	-	medial wall (2)	1 subject (bilateral)	Dental ectopia may occur in antrumand present
Jendi et al. [34]	Indian J Otolaryngol Head Neck Surg	Case report	24 yo	nasal discharge and heaviness	-	Exodontia	Intraoral Caldwell Luc procedure	Postoperative period unremarkable.	-	Lateral nasal wall	1 subject	Less invasive transnasal endoscopic shaver-assisted technique has been advocated for the retrieval of any foreign body
Ramanojam et al. [35]	Ann Maxillofac Surg	Case series	Range 21–32 yo	Pain and swelling cheek, pain ATM joint (1), Asymptomatic (1)	dentigerous cyst (1)	Exodontia	Caldwell-Luc procedure	Postoperative period unremarkable (5). hypoaesthesia of infraorbital nerve (1)	-	(a) Medial wall	6 subjects (6 cases)	The endoscopic techniques are being used for removal of an intranasal ectopic tooth
Mohan et al. [36]	Natl J Maxillofac Surg	Case report	28 yo	Pain and swelling, purulent rhinorrhea	-	Exodontia	Caldwell-Luc procedure	Postoperative period unremarkable.	-	Superomedial wall	1 subject	If untreated, it has the tendency to form a cyst or tumor and/or the lesion may cause perforation of the orbital floor and obliteration of the nasal cavity
Thakur et al. [37]	BMJ Case Rep	Case report	25 yo	Pain and swelling, purulent rhinorrhea		Exodontia	Caldwell-Luc procedure	Postoperative period unremarkable.	-	Lateral- Inferior wall	1 subject	It is important as certain antral diseases like dentigerous cyst, odontogenic keratocyst may co-exist with an ectopic molar
Demirtas et al. [38]	J Craniofac Surg	Case report	19 yo	pain, discomfort, and fullness in the right cheek	dentigerous cyst	Lesion enucleation/exodontia	First marsupialization, second enucleation with Caldwell-Luc approach (2 stage surgery)	Postoperative period unremarkable.	-	Floor of the orbit	1 subject	Early diagnosis and treatment of these lesions allow a reduced patient’s morbidity
Srinivasa Prasad et al. [39]	Indian J Dent Res	Case report	45 yo	recurrent purulent rhinorrhea	dentigerous cyst	Lesion enucleation/exodontia	Caldwell-Luc procedure	Postoperative period unremarkable.	-	Postero-medial wall	1 subject	The treatment of an ectopic tooth in the maxillary sinus is usually removal
AlKhudair et al. [40]	Int J Surg Case Rep	Case report	19 yo	recurrent sinusitis	dentigerous cyst	Lesion enucleation/exodontia	endoscopic-assisted middle meatal antrostomies surgery	Postoperative period unremarkable.	-	(a) Lateral wall (b) Medial wall	1 subject (bilateral)	The endonasal endoscopic approach is a minimally invasive approach
Aydın et al. [41]	Balkan Med J	Case report	21 yo	Sinus infection, oro-antral fistula	Osteoma	Lesion enucleation/exodontia	endonasal endoscopic uncinectomy and Caldwell-Luc maxillary antrostomy	recurrent oroantral fistula.	-	Posterior wall	1 subject	A pediatric maxillofacial trauma might cause the eruption of the tooth
Almomen et al. [42]	J Surg Case Rep	Case report	-	Sinusitis, nasal discharge	dentigerous cyst	Lesion enucleation/exodontia	transnasal endoscopic approach (TEA)	Postoperative period unremarkable.	-	medial wall	1 subject	The endonasal endoscopic approach is a direct minimally invasive procedure
Buyukkurt et al. [43]	Oral Surg Oral Med Oral Pathol Oral Radiol Endod	Case series	Range 19–32 yo	Swelling, pain	dentigerous cyst	Lesion enucleation/exodontia	Caldwell-Luc procedure	Postoperative period unremarkable.	-	(a) Latero-inferior wall (1) (b) Postero-inferior wall (1) (c) Lateral wall (1)	3 subjects (3 cases)	Transnasal extraction may be attempted if the tooth is small and near the ostium
Bodner et al. [44]	J Laryngol Otol	Case series	-	Swelling, pain, acurte sinusitis	dentigerous cyst	Lesion enucleation/exodontia	Caldwell-Luc procedure	Postoperative period unremarkable.	-	(a) Inferior-lateral wall (3) (b) Lateral wall (7) Supero-llateral wall	12 subjects	CT is useful for diagnosis and treatment planning of teeth in the maxillary antrum
Baykul et al. [45]	Auris Nasus Larynx	Case series	Range 15–52 yo	Swelling, pain, acurte sinusitis	dentigerous cyst (1)	(a) radiology follow-up (2) (b) lesion enucleation/exodontia (7)	Endoscopyc assisted surgery removal	Postoperative period unremarkable.	-	Postero-lateral wall	9 subjects (4 cases of ectopic 3rd molar)	Ectopic tooth in the maxillary sinus may lead to the misdiagnosis of sinusitis
Elmorsy et al. [46]	F1000Res	Case report	13 yo		dentigerous cyst	Lesion enucleation/exodontia	Intraoral Caldwell Luc procedure	Postoperative period unremarkable. the bone requiring longer time to form and be detected radiographically	−500 mg amoxicillin/8 h/5 days −325 mg acetaminophen/4 h/day −4 mg betamethasone	posterosuperior wall	1 subject	Asymptomatic cases should be managed with a similar protocol due to their tendency to form cysts or malignancies.

## Data Availability

All data generated or analyzed during this study are included in this published article.
